# Pedestrian and Overall Road Traffic Crash Deaths — United States and 27 Other High-Income Countries, 2013–2022

**DOI:** 10.15585/mmwr.mm7408a2

**Published:** 2025-03-13

**Authors:** Rebecca B. Naumann, Bethany A. West, Vaughn Barry, Sarah Matthews, Robin Lee

**Affiliations:** ^1^Division of Injury Prevention, National Center for Injury Prevention and Control, CDC; ^2^Division of Violence Prevention, National Center for Injury Prevention and Control, CDC.

**Summary**

**What is already known about this topic?**

U.S. road traffic crashes cause more than 40,000 deaths annually. Pedestrians are disproportionately affected.

**What is added by this report?**

During 2013–2022, U.S. traffic-related death rates increased a relative 50.0% for pedestrians and 22.5% overall, compared with those in 27 other high-income countries, where they declined a median of 24.7% and 19.4%, respectively. Across countries, U.S. pedestrian death rates were highest overall and among persons aged 15–24 and 25–64 years.

**What are the implications for public health practice?**

Pedestrian and overall road traffic deaths remain higher in the United States than in other high-income countries Increased adoption of evidence-based strategies to reduce these deaths, such as the Safe System approach which focuses on structural and policy changes, such as protected walkways and safe crossings, consistent street lighting, and speed management policies, might help reduce traffic deaths.

## Abstract

Road traffic deaths are preventable but remain a major public health problem. Crashes cause more than 40,000 deaths annually in the United States, and traffic-related pedestrian deaths have increased rapidly. To examine change in pedestrian and overall traffic death rates (deaths per 100,000 population) within an international context, CDC analyzed 2013–2022 data from the United States and 27 other high-income countries in the International Road Traffic and Accident Database, as well as early 2023 U.S. estimates. Between 2013 and 2022, U.S. pedestrian death rates increased 50% (from 1.55 to 2.33 per 100,000 population), while other countries generally experienced decreases (median decrease = 24.7%). During this period, overall U.S. traffic death rates increased 22.5% (from 10.41 to 12.76), but decreased by a median of 19.4% in 27 other high-income countries. Among all countries examined, the United States had the highest pedestrian death rates overall and among persons aged 15–24 and 25–64 years. Projected 2023 U.S. estimates suggest a potential decline in pedestrian (2%) and overall traffic (4%) deaths, compared with those in 2022. Accelerated adoption of a Safe System approach, focused on creating safer roadways and vehicles, establishing safer speeds, supporting safer road users, and improving post-crash care, can help reduce U.S. pedestrian and overall traffic deaths.

## Introduction

More than 40,000 lives are lost annually in the United States because of road traffic crashes, and traffic-related pedestrian deaths have increased rapidly over the last several years (*1*). In 2022, pedestrian deaths reached their highest number (7,522) in 41 years (*1*). Examining domestic and international trends in pedestrian and overall road traffic deaths can help guide and prioritize U.S. traffic safety efforts. This study compares pedestrian and overall road traffic death rates in the United States and 27 other high-income countries during 2013–2022 and, given that the data are not yet final, examines projected 2023 U.S. estimates based on crash reports. In addition, CDC examined age-related disparities in pedestrian death rates across countries.

## Methods

### Data Source

CDC obtained 2013–2022 road traffic death data from the International Transport Forum’s International Road Traffic and Accident Database (IRTAD).* IRTAD contains standardized and validated annual road traffic death and population data from 35 participating countries. The United States provides road traffic death data from the Fatality Analysis Reporting System (FARS), a census of crashes on U.S. public roadways that result in a death within 30 days of the crash.^†^ This analysis included data from all 28 high-income countries^§^ with populations of >1 million persons that provided data to IRTAD and had no major changes in how data were reported during this period.^¶^^,^** At the time of this investigation and publication date, 2023 and FARS data were not yet final, so projected estimates of 2023 U.S. pedestrian and overall road traffic deaths calculated in May 2024 by the U.S. Department of Transportation are included here to provide a more recent description of possible U.S. trends.^††^

### Data Analysis

CDC analyzed country-specific pedestrian and overall road traffic^§§^ deaths and death rates (deaths per 100,000 population) from 2013 through 2022 and calculated absolute and relative percentage changes in rates by country, as well as mean and median rates and rate changes, with and without the United States included. Country-specific 5-year annual average pedestrian death rates were calculated by age group (0–14, 15–24, 25–64, and ≥65 years) using 2018–2022 data;^¶¶^ the 5 most recent years of data (2018–2022) were combined to support estimate stability, given small annual counts and high between-year variability by age group for some countries. Analyses were descriptive and conducted using Excel. This activity was reviewed by CDC, deemed not research, and conducted consistent with applicable federal law and CDC policy.***

## Results

### Pedestrian Road Traffic Crash Deaths

**FIGURE 1:**
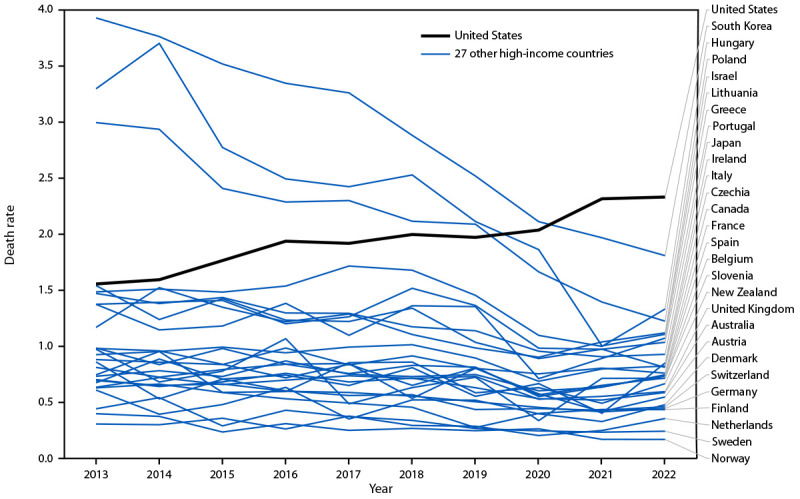
Pedestrian death rates,* by country — United States and 27 other high-income countries, 2013–2022^†^ * Deaths per 100,000 population. ^†^ Data from the International Transport Forum’s International Road Traffic and Accident Database.

During 2013–2022, U.S pedestrian death rates increased a relative 50.0% (from 1.55 to 2.33 deaths per 100,000 population), while most other countries experienced decreases (median relative decline = 24.7%; IQR = −45.0% to −10.4%) (Figure 1) (Table). By 2022, pedestrian death rates in all other 27 countries were lower than the U.S. rate, with the U.S. pedestrian death rate (2.33) approximately three times the median rate of the 27 other countries (0.73). There were 2,857 more pedestrian deaths in the U.S. in 2022 than in 2013, and 3,071 fewer pedestrian deaths in 2022 than in 2013 in the 27 other countries.

**TABLE:** Pedestrian and overall road traffic deaths and death rates,* by country — United States and 27 other high-income countries, 2013 and 2022^†^

Country	Pedestrian traffic deaths						Overall road traffic deaths					
	2013		2022		10-year change in rate^§^		2013		2022		10-year change in rate^§^	
	No. of deaths	Rate*	No. of deaths	Rate*	Absolute change	Relative % change	No. of deaths	Rate*	No. of deaths	Rate*	Absolute change	Relative % change
**Australia**	**162**	**0.70**	**152**	**0.58**	**−0.12**	**−16.6**	**1,185**	**5.12**	**1,111**	**4.27**	**−0.85**	−16.6
Austria	83	0.98	49	0.55	−0.44	−44.4	455	5.38	370	4.12	−1.26	−23.5
Belgium	109	0.98	83	0.71	−0.26	−27.0	764	6.86	540	4.65	−2.21	−32.2
Canada	309	0.88	295	0.76	−0.12	−13.8	1,951	5.55	1,934	4.97	−0.58	−10.5
Czechia	162	1.54	85	0.81	−0.73	−47.5	654	6.22	527	5.01	−1.21	−19.4
Denmark	34	0.61	28	0.48	−0.13	−21.4	191	3.41	154	2.62	−0.79	−23.1
Finland	34	0.63	24	0.43	−0.19	−31.0	258	4.75	189	3.41	−1.35	−28.3
France	465	0.73	488	0.74	0.01	1.8	3,268	5.13	3,267	4.98	−0.15	−3.0
Germany	557	0.69	368	0.44	−0.25	−36.1	3,339	4.15	2,788	3.35	−0.80	−19.2
Greece	151	1.37	112	1.07	−0.30	−22.0	879	7.99	654	6.25	−1.74	−21.7
Hungary	147	1.48	129	1.33	−0.15	−10.3	591	5.96	535	5.52	−0.44	−7.4
Ireland	31	0.67	43	0.85	0.18	26.4	188	4.08	155	3.06	−1.02	−24.9
Israel	94	1.17	108	1.12	−0.05	−4.2	309	3.83	351	3.63	−0.20	−5.3
Italy	551	0.92	485	0.82	−0.10	−11.0	3,401	5.70	3,159	5.35	−0.35	−6.1
Japan	1,871	1.47	1,157	0.93	−0.54	−37.0	5,165	4.06	3,216	2.57	−1.48	−36.6
Lithuania	98	3.30	31	1.10	−2.19	−66.5	258	8.68	120	4.28	−4.40	−50.7
Netherlands	51	0.30	62	0.35	0.05	16.0	476	2.84	655	3.72	0.89	31.3
New Zealand	33	0.74	34	0.66	−0.08	−10.6	252	5.67	375	7.33	1.65	29.2
Norway	20	0.40	9	0.17	−0.23	−58.1	187	3.70	116	2.14	−1.56	−42.2
Poland	1,140	3.00	460	1.22	−1.77	−59.2	3,357	8.82	1,896	5.04	−3.78	−42.9
Portugal	144	1.37	107	1.03	−0.34	−24.7	637	6.07	618	5.97	−0.10	−1.7
Slovenia	20	0.97	15	0.71	−0.26	−26.7	125	6.07	85	4.03	−2.04	−33.6
South Korea	1,982	3.93	933	1.81	−2.12	−54.0	5,092	10.10	2,735	5.30	−4.80	−47.5
Spain	378	0.81	348	0.73	−0.08	−9.3	1,680	3.60	1,746	3.68	0.09	2.4
Sweden	42	0.44	25	0.24	−0.20	−45.5	260	2.72	227	2.17	−0.55	−20.1
Switzerland	69	0.86	40	0.46	−0.40	−46.7	269	3.35	241	2.76	−0.59	−17.6
United Kingdom	405	0.63	401	0.59	−0.04	−6.1	1,770	2.76	1,766	2.61	−0.15	−5.4
United States	4,911	1.55	7,768	2.33	0.78	50.0	32,893	10.41	42,514	12.76	2.35	22.5
**Overall Measures**												
Mean (incl United States)	502	1.18	494	0.82	−0.36	−22.7	2,495	5.46	2,573	4.48	−0.98	−16.2
Mean (excl United States)	339	1.17	225	0.77	−0.40	−25.4	1,369	5.28	1,094	4.18	−1.10	−17.7
Median (incl United States)	146	0.90	108	0.74	−0.20	−23.3	646	5.26	579	4.20	−0.79	−19.3
Median (excl United States)	144	0.88	107	0.73	−0.20	−24.7	637	5.13	540	4.12	−0.80	−19.4

**Abbreviations**: excl = excluding; incl = including.

* Deaths per 100,000 population.

^†^ Data from the International Transport Forum’s International Road Traffic and Accident Database.

^§^ Absolute (2022 rate − 2013 rate) and relative % (([2022 rate − 2013 rate] / 2013 rate) x 100) changes displayed are based on unrounded rates and therefore might differ slightly from calculations based on rounded rates.

### Overall Road Traffic Crash Deaths

During this 10-year period, overall U.S. road traffic death rates increased a relative 22.5%, from 10.41 to 12.76 deaths per 100,000. In the 27 other countries, overall road traffic death rates generally decreased (median decrease = 19.4%; IQR = −30.3% to −5.7%). Projected 2023 estimates obtained from May 2024 U.S. Department of Transportation calculations indicate small relative declines in overall U.S. traffic deaths (4%) and pedestrian deaths (2%) between 2022 and 2023.

### Characteristics of Pedestrians Killed in Road Traffic Crashes

**FIGURE 2:**
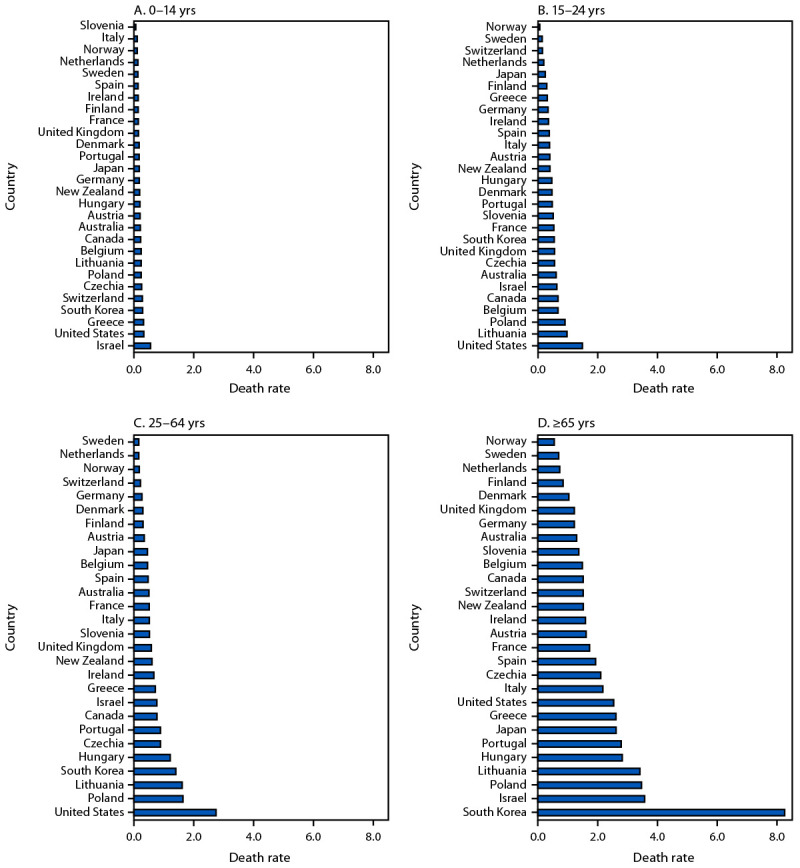
Five-year annual average pedestrian road traffic crash death rates* among persons aged 0–14 (A), 15–24 (B), 25–64 (C), and ≥65 years (D) — United States and 27 other high-income countries, 2018–2022^†^^,^^§^ **Abbreviation**: IRTAD = International Transport Forum’s International Road Traffic and Accident Database. * Deaths per 100,000 population. ^†^ Data from IRTAD. ^§^ 2022 pedestrian death counts by age group for Canada were unavailable from IRTAD and could not be obtained from country-specific websites; therefore, Canada’s pedestrian death rates, by age group, represent 4-year average rates, using 2018–2021 data.

During 2018–2022, across most countries examined, the highest pedestrian death rates were observed in adults aged ≥65 years, with the highest rates in South Korea (Figure 2). Compared with other countries, the United States had the highest pedestrian death rates among persons aged 15–24 and 25–64 years, and the second highest rate among children aged 0–14 years.

## Discussion

During 2013–2022, U.S. road traffic crash pedestrian death rates increased, while rates in many other high-income countries decreased. In 2022, the U.S. pedestrian death rate was higher than that in all 27 included high-income countries and approximately three times the median rate in these countries. Moreover, whereas the overall U.S. road traffic death rate increased as well during this time, the pedestrian death rate increase was approximately twice as large. These findings update previous surveillance findings indicating that the United States has often lagged behind road safety progress of other high-income countries (*2*,*3*). However, previous work has not analyzed international changes in pedestrian deaths, whose death rates have long been increasing in the United States (*1*).

There are many possible contributors to the increases in U.S. pedestrian and overall road traffic death rates (*4*), including a changing mix of vehicles on U.S. roadways and changing dimensions of these vehicles (*5*). The proportion of taller and heavier vehicles with poor visibility (e.g., sport utility vehicles [SUVs] and pickup trucks) has increased, and the physical characteristics of these vehicles have become larger over time (e.g., heavier overall weight and higher bumpers), making them more likely to be involved in certain types of crashes and to result in death when crashes occur (*5*). SUVs, vans, and pickup trucks accounted for 79% of new U.S. leases and vehicle sales in 2022, while the proportion of smaller vehicles (e.g., sedans) declined from 50% of new vehicles in 2012 to 21% in 2022 (*6*). Compared with passenger cars, SUVs and pickup trucks are more likely to strike pedestrians during certain maneuvers (e.g., turning), and pedestrians are 50%–100% more likely to be killed when they are in a crash involving a SUV or pickup truck (*5*).

High-speed and complex, multilane roadways (e.g., arterial roadways) also are associated with increased U.S. pedestrian deaths (*7*). Many of these roadways are characterized by increased crash risk linked to conflicting goals of providing immediate access to key commercial destinations (e.g., stores and restaurants), while also seeking to move vehicles at high travel speeds (*7*). Several other countries use different roadway design strategies, including prioritizing land use and safe movement by sustainable travel modes (i.e., walking, cycling, and transit) (*8*). Further, the increase in the number of persons living below the poverty line, particularly in U.S. suburban communities with fewer transportation options and less access to safe pedestrian infrastructure, could contribute to higher U.S. pedestrian death rates (*9*).

Certain age groups are disproportionately affected by pedestrian deaths including older adults, who generally experience the highest rates. Several factors, including walking speed, vision impairment, and distance judgement might increase risk among older adults; however, design solutions, such as lengthening pedestrian intervals, narrowing crossing distance, and adequate lighting, could mitigate this risk (*10*). These same strategies can help prevent crashes among pedestrians of all ages, and more widespread adoption of these and other design strategies are needed, given high U.S. pedestrian death rates across age groups. In addition, further research is needed on countries with high pedestrian death rates but notable recent progress. For example, although South Korea had the highest older adult pedestrian death rate, the rate decreased by 61% (from 15.79 to 6.19) during 2013–2022.

The Safe System approach, grounded in public health principles, is a framework for building in layers of evidenced-based prevention strategies to ensure that no crash results in death or serious injury.^†††^ The approach also stresses a need to minimize high speeds and impact forces through these layers of protection, a principle particularly important for protecting pedestrians. Application of the Safe System approach includes implementing population-level strategies like creating environments that encourage travel at safe speeds (e.g., lane narrowing), separating different types of road users (e.g., protected walkways), and supporting safe road user behaviors (e.g., ignition interlock devices to prevent impaired individuals from operating vehicles).

Several countries have reduced road traffic death rates over recent decades with adoption of the Safe System approach.^§§§^ The U.S. federal government cemented its adoption of the Safe System approach in the 2022 National Roadway Safety Strategy.^¶¶¶^ In addition, the international community has recognized the importance of this approach, calling for increased implementation of a Safe System approach during a second Decade of Action for Road Safety (2021–2030).**** While projected 2023 estimates demonstrate a small potential reduction in U.S. pedestrian deaths, widespread implementation of a Safe System approach in the United States could help accelerate progress.

### Limitations

The findings in this report are subject to at least two limitations. First, population counts were used for rate denominators in this analysis, rather than an exposure-based measure (e.g., vehicle miles or kilometers traveled). Exposure-based data are not available for pedestrians across all countries. However, given sprawl and land use patterns have been associated with increasing U.S. road traffic death rates,^††††^ population-based estimates provide a useful measure that does not adjust for potential contributors to the problem (e.g., sprawl). Second, rates are not age-adjusted, and country-specific population age distributions could influence rankings and comparisons. However, overall findings of disproportionate growth in the U.S. pedestrian death rate, compared with other countries, would not be impacted.

### Implications For Public Health Practice

The U.S. pedestrian road traffic death rate has rapidly increased over the last several years, representing a contrast to decreasing trends in many other high-income countries. Whereas 2023 U.S. projections indicate a potential reduction in pedestrian deaths, increased and more widespread application of the Safe System approach could help accelerate and sustain progress. Public health practitioners play a critical role in Safe System implementation through use of health-related data sources and prevention frameworks to guide and prioritize strategy implementation, in building multidisciplinary coalitions to support widespread adoption of population-level road traffic injury prevention strategies, in co-designing interventions with partners and communities, and in rapidly evaluating interventions to guide progress. Numerous strategies exist to create preventive systemwide redundancies, consistent with a Safe System approach, to reduce pedestrian and overall road traffic deaths, including protected walkways and safe crossings, consistent street lighting, and speed management policies.

## Footnotes

* IRTAD data and annual reports are available at https://www.itf-oecd.org/irtad-road-safety-database and https://www.itf-oecd.org/irtad-publications. Data were current with respect to those reported to the International Transport Forum (ITF) as of June 2024. Thirty-five countries report data to IRTAD, and the following 28 are included in this analysis: Australia, Austria, Belgium, Canada, Czechia, Denmark, Finland, France, Germany, Greece, Hungary, Ireland, Israel, Italy, Japan, South Korea, Lithuania, Netherlands, New Zealand, Norway, Poland, Portugal, Slovenia, Spain, Sweden, Switzerland, the United Kingdom, and the United States.

† FARS captures information on road traffic crash deaths from a variety of sources, including police crash reports, medical examiner reports, emergency medical services reports, and state vehicle registration and driver’s licensing files. https://www.nhtsa.gov/crash-data-systems/fatality-analysis-reporting-system

§ The definition of high-income derives from the World Bank’s classification scheme and is based on gross national income per capita. https://datatopics.worldbank.org/world-development-indicators/the-world-by-income-and-region.html

¶ Among the 35 countries that report data to IRTAD, seven were not included in this analysis (with reasons for exclusion in parentheses): Argentina (upper-middle–income), Chile (definition for road traffic crash deaths changed in 2019, which created a trend break), Columbia (upper-middle–income), Costa Rica (upper-middle–income), Iceland (population <1 million), Luxembourg (population <1 million), and Serbia (upper-middle–income).

** Data were supplemented from additional sources to ensure complete 2022 death and population data. Data from other sources included final 2021 and 2022 U.S. overall road traffic, pedestrian, and pedestrian by age group deaths from FARS and 2022 overall road traffic, pedestrian, and pedestrian by age group deaths for Greece from the Hellenic Statistical Authority (https://www.statistics.gr/). 2022 population estimate data were obtained from relevant census sources for Canada (https://www12.statcan.gc.ca/census-recensement/index-eng.cfm), the United Kingdom (https://www.ons.gov.uk/), and the United States (https://www.census.gov/).

†† 2023 early estimate data for FARS are provided by the National Highway Traffic Safety Administration data; additional data on estimates are available at https://crashstats.nhtsa.dot.gov/Api/Public/ViewPublication/813581.

§§ Overall road traffic deaths included deaths to vehicle occupants, motorized two-wheelers, pedestrians, and cyclists, as well as other road user types. Pedestrian deaths in IRTAD include persons on foot or personal conveyances.

¶¶ Age groups are predetermined by ITF, and pedestrian sex data were not available. In addition, 2022 pedestrian death counts by age group for Canada were unavailable from IRTAD and could not be obtained from country-specific websites; therefore, Canada’s pedestrian death rates by age group represent 4-year average rates, using 2018–2021 data.

*** 45 C.F.R. part 46, 21 C.F.R. part 56; 42 U.S.C. Sect. 241(d); 5 U.S.C. Sect. 552a; 44 U.S.C. Sect. 3501 et seq.

††† https://publichealth.jhu.edu/sites/default/files/2023-03/recommendations-of-the-safe-system-consortium.pdf

§§§ https://www.roadsafety.unc.edu/wp-content/uploads/2022/09/CSCRS_R3_Final-Report-2022.pdf

¶¶¶ https://www.transportation.gov/NRSS

**** https://press.un.org/en/2024/ga12609.doc.htm; https://www.who.int/publications/m/item/global-plan-for-the-decade-of-action-for-road-safety-2021-2030.

††††
https://ajph.aphapublications.org/doi/full/10.2105/AJPH.93.9.1541
